# Acids in Disorders of the Bowels

**Published:** 1855-03

**Authors:** 


### ACIDS IN THE TREATMENT, PROPHYLACTIC AND REMEDIAL, OF EPIDEMIC DISORDERS OF THE BOWELS.

An interesting paper on this subject was read before the Epidemiological Society, July 3, 1854, by J. H. Tucker, Esq. The author commenced by alluding to the remarkable, but well-established fact, that in 1849 the cider districts of Herefordshire, Somersetshire, and part of Devonshire, were, to a great extent, exempt from the epidemic ravages of cholera, while the disease was raging around. Upon farther inquiry, it was ascertained that this exemption was confined a good deal to those individuals who drank cider as a common beverage, and that those who partook of malt liquor occasionally suffered. Also, in some parts of France and Normandy, more particularly where cider is the common beverage, cholera is seldom known to exist; and farther, Switzerland was reported to have been free from its visitation.

From these and other facts in proof of the prophylactic power of cider, the author expressed his opinion that other vegetable acids would be found of service, such as lemon-juice, orange-juice, and sour wines made from grapes, or even from gooseberries. And as it would be found impossible to supply the demand for a sufficient quantity of pure cider, *vinegar* might be found a useful substitute in case of another outbreak of cholera, provided that it could be obtained in a state of purity. In confirmation of this view of the sanative and medicinal virtues of vinegar, the author quoted Hippocrates, who "employed white vinegar medicinally"—Plutarch and Livy, who refer to the use of vinegar by Hannibal, in his passage over the Alps, when he is said to have "softened the rocks with fire and vinegar," an operation which the author facetiously regarded as rather metaphorical than chemical, as the vinegar, swallowed by the troops, probably sustained their strength, and thus in effect softened the asperities of their rough way. He also quoted from Roman history the story that "Scipio Africanus is said to have gained a great battle with a few skins of vinegar," the troops refusing to march until the general had obtained a supply. Cæsar was also reported to mention in his Commentaries the supply of vinegar to the troops; and Mr. Tucker remarked that the drink of the Romans in all their campaigns was vinegar and water, and, sustained by that beverage, they conquered the world. Modern authors, (Sir John Pringle, Sir Gilbert Blane, and others) were also quoted in proof of the antiseptic and medicinal qualities of vinegar. The author then proceeded to show that acid drinks were not only preventive, but remedial in epidemic disorders of the bowels. Cases were related, in which not only persons were exempt from attacks of cholera raging around them, who drank large draughts of cider, but a case of severe cholera was also related, which yielded to the diluted juice of sour apples. The efficacy of the *Mineral Acids*, especially the sulphuric, in diarrhœa, and especially in choleraic diarrhœa, was also advocated by reference to numerous facts and authorities. He also referred to some established facts connected with the spread of epidemic dysentery in the army, showing the efficacy of vegetable acids in that disease.

Mr. Tucker suggested a necessary caution relative to the use of the unwholesome substitute for vinegar commonly sold in the shops.

The discussion which followed the reading of the paper, elicited many facts in confirmation of the author's views; and, as to the efficacy of sulphuric acid largely diluted with water, in choleraic diarrhœa, there was not a dissentient voice.—*Lancet.*

